# First person – Elisa Thoral

**DOI:** 10.1242/bio.060392

**Published:** 2024-03-25

## Abstract

First Person is a series of interviews with the first authors of a selection of papers published in Biology Open, helping researchers promote themselves alongside their papers. Elisa Thoral is first author on ‘ The relationship between mitochondrial respiration, resting metabolic rate and blood cell count in great tits’, published in BiO. Elisa is a postdoctoral researcher in the lab of Dr Andreas Nord at Lund University, Lund, Sweden, investigating I am interested in the effects of environmental parameters, such as temperature and diet, on animals’ performance at both the organismal and cellular level.


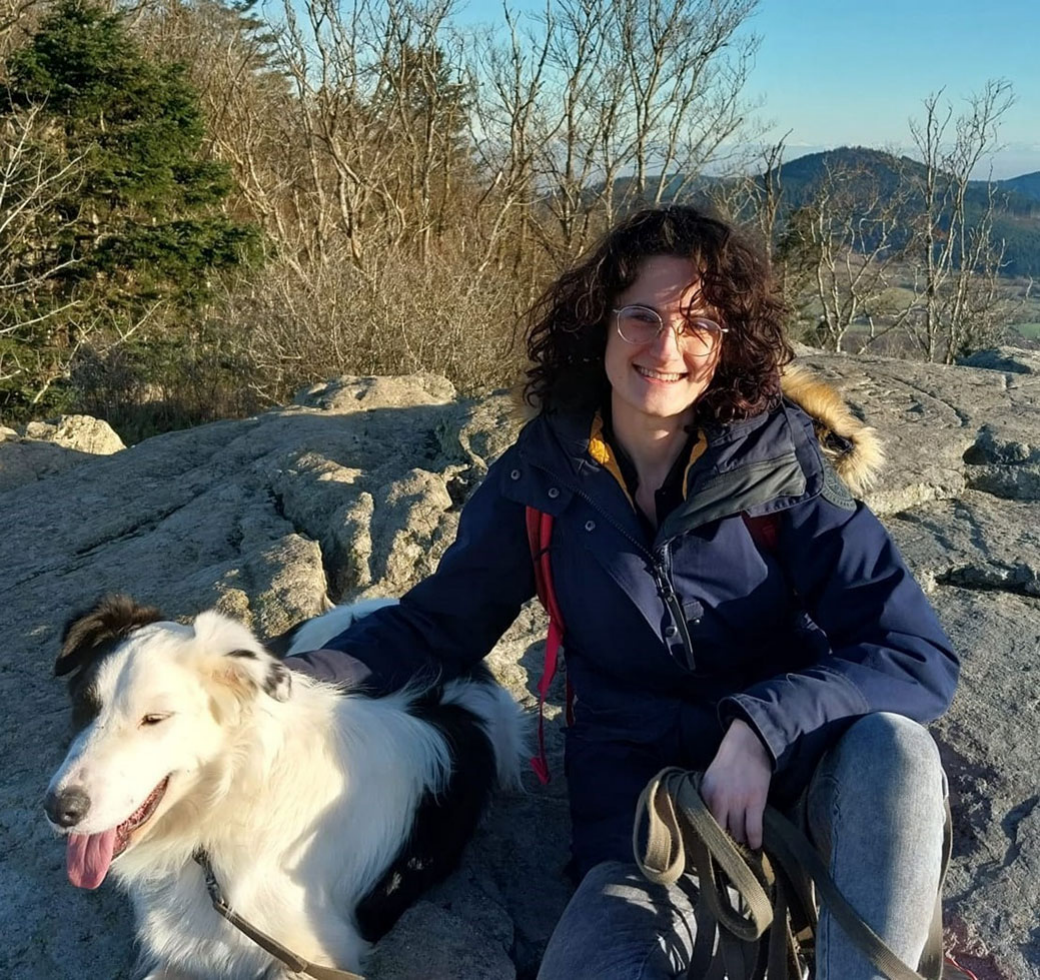


**Elisa Thoral**

**Describe your scientific journey and your current research focus**

I defended my PhD in 2021 in Lyon, France. I was interested in the physiological consequences of environmental stressors in fish. Specifically, I measured swimming performance and mitochondrial metabolism in fish muscle in response to variation in temperature, oxygen and food availability. I'm currently doing a postdoctoral project in Lund, Sweden, investigating how temperature fluctuations and diet manipulations affect temperature tolerance and metabolic rates at both organismal and cellular levels in different life stages of birds.

**Who or what inspired you to become a scientist?**

I think that it is my curiosity about how the world around us works, from the cell to the universe, that drove me to become a scientist.

**How would you explain the main finding of your paper?**

Our study shows that, to a certain extent, the rate at which great tits spend energy can be explained by how much oxygen their blood cells consume.

Our study shows that, to a certain extent, the rate at which great tits spend energy can be explained by how much oxygen their blood cells consume.

**What are the potential implications of this finding for your field of research?**

These results show that the link between individual and cellular metabolic rate is not necessarily evident, depending on both the studied tissues and how these were treated before the measurements. Moreover, normalizing mitochondrial respiration by the total cell count does not seem to be an appropriate method in the case of bird blood.

**Figure:**
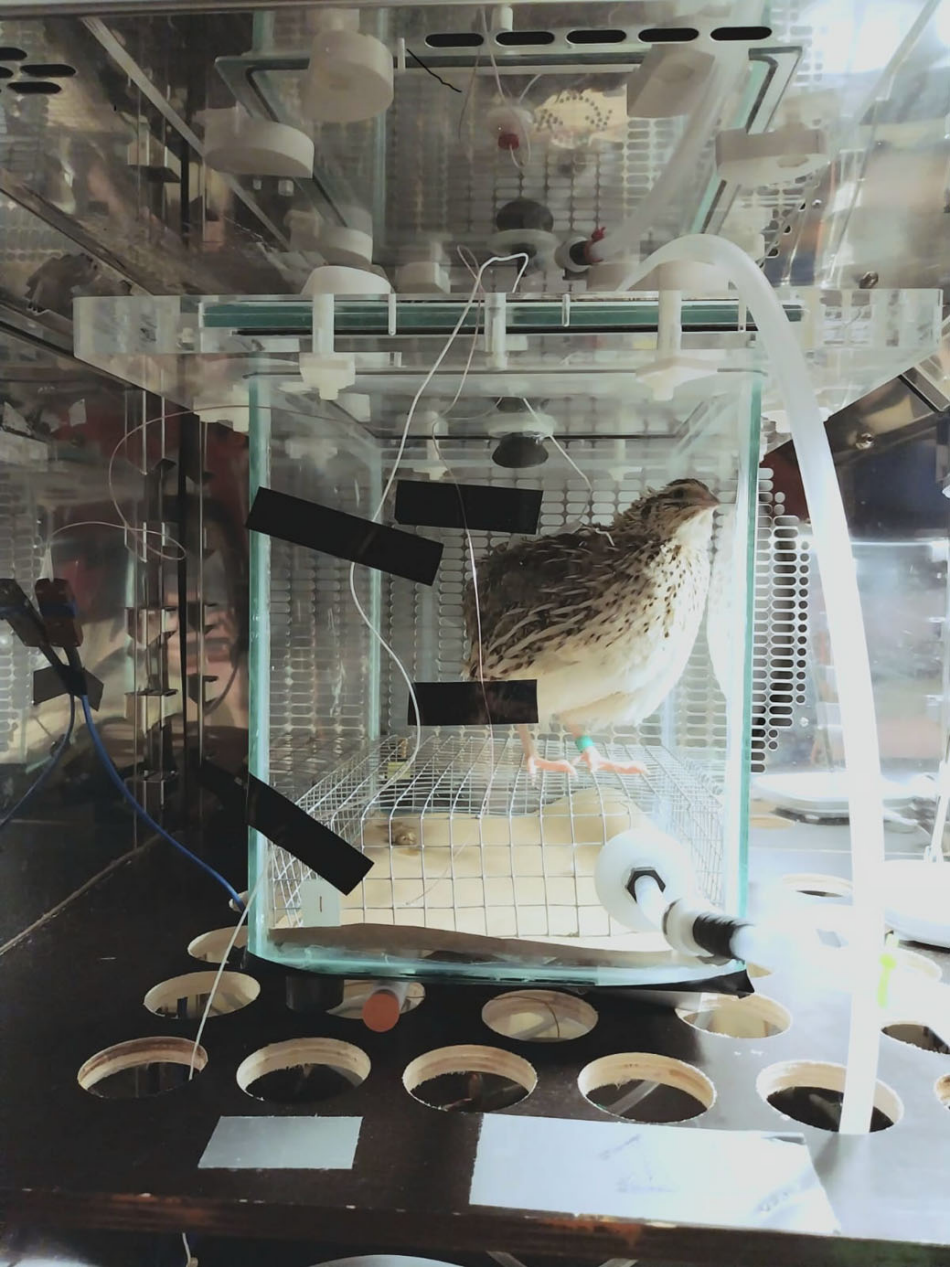
Resting metabolic rate and the shivering response measured at different temperatures in Japanese quail

**Which part of this research project was the most rewarding?**

These data are the first that I analyzed at the start of my postdoctoral project, and this article is therefore the first one to come out of the work carried out during my postdoc.

**What do you enjoy most about being an early-career researcher?**

I enjoy being able to do the research I want to do, while having the chance to get advice from my supervisors.

**What piece of advice would you give to the next generation of researchers?**

Communication between you and your colleagues and supervisors is the key to succeed in research.

**What's next for you?**

I am planning to continue to work as a researcher, through future postdoc projects and hopefully a permanent position!
